# Effect of continuous positive airway pressure associated to exercise on the breathing pattern and heart rate variability of patients undergoing coronary artery bypass grafting surgery: a randomized controlled trial

**DOI:** 10.1590/1414-431X2021e10974

**Published:** 2021-08-20

**Authors:** C.B.F. Pantoni, L. Di Thommazo-Luporini, R.G. Mendes, F.C.R. Caruso, V. Castello-Simões, D. Mezzalira, A. Borghi-Silva

**Affiliations:** 1 Departamento de Gerontologia, Universidade Federal de São Carlos, São Carlos, SP, Brasil; 2 Departamento de Fisioterapia, Universidade Federal de São Carlos, São Carlos, SP, Brasil

**Keywords:** CPAP, Breathing pattern, Coronary artery bypass grafting surgery, Cardiac rehabilitation, Inpatient

## Abstract

Continuous positive airway pressure (CPAP) has been used to improve gas exchange and diaphragmatic function, among others benefits. Moreover, it can be used to increase exercise tolerance and positively influence ventilatory function and breathing pattern (BP) during exercise. However, there is no information about the long-term effects of CPAP, as an adjunct to an inpatient cardiac rehabilitation (CR) program, on BP and heart rate variability (HRV) of patients after coronary artery bypass grafting surgery (CABG). Twenty patients were allocated to receive, after randomization, standard inpatient CR without CPAP (control group - CG) or CR with CPAP between 10 to 12 cmH_2_O (CPAP group - CPG) associated with the exercises. Participants were assessed preoperatively and on the discharge day, in the sitting rest position. Outcome measurements included BP variables, collected by respiratory inductive plethysmography, and HRV, collected by polar precision performance. The CPG presented lower values of percent rib cage inspiratory and expiratory contributions to tidal volume (%RCi and %RCe) at discharge time, compared to CG. No statistical differences between groups were observed for HRV variables and both groups presented lower values of these indices, compared to preoperative ones. In this context, the patients who received CPAP throughout the whole rehabilitation program were discharged with a better BP, which could indicate more synchronized breathing. CPAP did not influence cardiac autonomic modulation in the long term.

## Introduction

Coronary artery bypass grafting (CABG) surgery continues to be the treatment of choice for patients with advanced and complex coronary artery disease (1,2), and it is indicated to improve myocardial function and reduce mortality associated to ischemia (3,4). However, the procedure and its complexity have been associated with some postoperative disorders. Decline of physical capacity (5), cardiac autonomic nervous system dysfunction (6,7), postoperative respiratory disorder, and lung function reduction have already been reported in patients after this type of surgery (8,9). Previously, we also identified a negative effect on patients' breathing pattern (BP), leading to an increase of thoracoabdominal asynchrony in the CABG postoperative period (10).

Several strategies of rehabilitation have been applied to these patients to prevent or minimize the decrease of functional capacity (11), the impact of surgery on cardiac autonomic modulation (7), and respiratory/pulmonary complications (12). In this context, our group was able to demonstrate a great benefit of early mobilization associated with chest physical therapy on cardiac autonomic modulation. In a previous study, Mendes et al. (13) showed that a short-term supervised physiotherapy exercise protocol applied during inpatient rehabilitation improves heart rate variability (HRV) at the time of discharge, compared to conventional respiratory exercises.

Among the different rehabilitation protocols applied after CABG (11), some adjuncts to physiotherapy have been used, such as the continuous positive airway pressure (CPAP), a modality of noninvasive ventilation, with a potential to improve cardiac autonomic modulation, gas exchange, diaphragmatic function, lung compliance, BP, and airway resistance, thus reducing the work of breathing (10,14).

In a study carried out by our group (10), acute application of CPAP was able to positively affect cardiac autonomic modulation and BP of patients after CABG. Furthermore, during the exercise, we demonstrated that CPAP, applied at the first day of walking, can increase exercise tolerance and positively influence ventilatory function and BP (15). However, in this last study, we identified the acute effect of CPAP during a single exercise. Considering that CPAP is a respiratory adjunct that improves gas exchange and diaphragmatic function and acutely positively affects BP, we aimed firstly to investigate if there is a long-term effect of CPAP on BP after inpatient cardiac rehabilitation (CR), that is, if the positive influence of CPAP on BP, previously observed only in the acute moment, can persist after concluding the rehabilitation program, measured during spontaneous breathing. Moreover, we also investigated if there is an effect of this adjunct on cardiac autonomic modulation.

In this context, the aim of our randomized controlled trial was to test the hypothesis that long-term use of CPAP associated with the exercises of a hospital rehabilitation program after CABG influences the BP, evaluated by respiratory inductive plethysmography (RIP), and cardiac autonomic modulation, evaluated by HRV.

## Material and Methods

### Study design

The present study is a prospective, randomized, controlled trial, conducted within the Coronary Unit and Cardiovascular Ward of a tertiary hospital and the methodological design was based on the CONSORT Statement (16). All the patients were instructed about the purpose and procedures of the study and informed consent was obtained prior to participation. The protocol applied followed the principles of the Declaration of Helsinki and was approved by the Human Research Ethics Committee of the University.

### Patients

We included patients of both genders, with clinical diagnosis of coronary artery disease with elective CABG surgery, median sternotomy incision, and interposition of a saphenous vein, internal thoracic artery, or radial artery grafts. Exclusion criteria were: emergent or other concomitant surgical approaches, valvular heart disease, previous cardiac surgery, unstable angina, use of an intra-aortic balloon pump or permanent pacemaker, chronic and acute disturbances in heart rhythm, chronic obstructive pulmonary disease, severe non-cardiac diseases, and/or incapacity to perform the protocol.

Preoperatively, a clinical cardiac assessment was conducted by a physician before the inclusion of patients in the study; additionally, demographic and anthropometry characteristics and clinical variables were recorded. At this point, patients were randomized (through a balanced randomization with a block size of four) to two different groups: with CPAP (CPG: continuous positive airway pressure group) or without CPAP (CG: control group) during the exercises applied in the postoperative period.

### Intervention

The inpatient CR was applied as described in a previous study (15) and consisted of a twice-daily supervised physiotherapy program of early mobilization, from the first postoperative day until hospital discharge (Table 1). For the patients allocated to the CG, all the physical exercises were performed during spontaneous breathing, and for the patients allocated to the CPG, the inpatient CR was the same plus a ventilatory support of CPAP (S8 Elite, Resmed, Australia) between 10 and 12 cmH_2_O, according to their tolerance, during all physical exercises. During the postoperative period, patients were monitored for adverse events in order to determine tolerance and progression of the protocol.

**Table 1:** Cardiovascular rehabilitation program applied to the groups from postoperative day one until hospital discharge.

Days	Description of protocol
1	Respiratory exercises: deep breathing from functional residual capacity to total lung capacity in three times of inspiration (1 set, 20 breaths); sustained respiration (5") at the end of inspiration (1 set, 20 breaths); bronchial hygiene maneuvers followed by coughing exercises. Physical exercise: bed inclined to 45°: active-assistive exercises of the lower/upper extremities; flexion and extension of ankles and wrists (5 sets, 10 reps).
2	Respiratory exercises: as in day 1. Physical exercises: sitting position to 90°; active-assistive exercises of extension and flexion of the shoulders, elbows, wrists, knees, and ankles and adduction and abduction of the hips (2 sets, 15 reps); orthostatic position (5′) as tolerated by the patient.
3	Respiratory exercises: as in day 2. Physical exercises: sitting position to 90°; active exercises as in day 2 (3 sets, 15 reps) and ambulation within the inpatient ward (5′).
4	Respiratory exercises: as in day 2. Physical exercises: sitting position to 90°; active exercises as in day 3 (3 sets, 15 reps) and ambulation within the inpatient ward (10′).
5	Respiratory exercises: as in day 2. Physical exercises: orthostatic position; active exercises as in day 4 (3 sets, 15 reps), ambulation within the inpatient ward (10′), and walk up and down flight of stairs (4 steps).

Inpatient cardiac rehabilitation consisted of a twice-daily supervised physiotherapy program.

### BP and HRV analysis

The BP and HRV variables were recorded at two different times: 1) preoperative situation (pre), for basal characterization, performed during 10 min in the sitting rest position; and 2) at discharge situation (post), during 10 min in the sitting rest position (before the initiation of the protocol). Care was taken to avoid any manipulations of the patients during recording.

BP signals were continuously measured with the RIP equipment, a multi-channel ambulatory monitor (LifeShirt System, Vivometrics Inc., USA) designed to register electrocardiogram and respiration. The respiration is registered by the upper ribcage and abdominal inductance plethysmography bands integrated in a garment, located at the levels of the nipples and umbilicus, respectively. A volumetric calibration was carried out with a fixed volume bag (800 mL) before initiating the data collection and the data were recorded with a portable device and downloaded to a computer into the specific program (VivoLogic, Vivometrics, USA) for analyses.

We carried out a quantitative calibration (fixed volume least squares calibration) to obtain the respiratory inductive plethysmographic sum signal for the absolute volume (mL). The breath-by-breath analysis was done during 30 breaths (chosen according to the most stable strength signal) in order to compare equal numbers of breaths from each individual in each situation and converted to mean values for statistical comparisons.

The BP analyses were assessed by volume measurements, with tidal volume (Vt) and minute ventilation (V_E_) and by thoracoabdominal coordination, represented by the percent rib cage inspiratory and expiratory contribution to Vt (%RCi and %RCe, respectively), which were obtained from the ratio of the inspiratory or expiratory volume related to the thoracic compartment by total tidal volume (that is, the sum of the thoracic and abdominal compartment), at the peak of inspiration or expiration, respectively (10).

The R-R interval (R-Ri) captured was reviewed by visual inspection using the Polar Precision Performance (Finland). A series containing sequential 300 R-Ri was analyzed using Kubios^®^ HRV analysis software 2.0 for Windows (MATLAB, version 2 beta, Finland). HRV was analyzed with linear statistical measures in time-domain and through nonlinear statistical measures. Mean of RR and heart rate (HR), standard deviation of all N-N normal intervals (SDNN), and the square root of the mean squared differences of successive RR (rMSSD) were computed as linear statistical measures in time-domain, in which the last two indices were representative of the global HRV and parasympathetic modulation, respectively (17). In addition, nonlinear statistical measures were calculated by standard deviation of Poincaré plot perpendicular to the line-of-identity (SD1), which indicates parasympathetic modulation (18).

### Outcome

Therefore, the primary outcome of this study was the BP at discharge time, during rest. The secondary outcome included HRV also at discharge time, during the rest position.

### Statistical analysis

Statistical analyses were performed by an investigator blinded to the experimental procedures. Analyses were performed using the SigmaPlot software 11.0 (Systat Software Inc., USA) and the Shapiro-Wilks test was used to investigate the data distribution. Paired and unpaired Student's *t-*tests were used to compare continuous variables within and between groups: age, anthropometry, RIP variables, HRV variables, and clinical data and the Fisher's exact test was used to compare categorical variables between groups: number of males, risk factors, and medications. We compared both groups at baseline to confirm that successful randomization had been achieved. All the tests were two-sided and a P-value <0.05 was considered statistically significant.

## Results

**Figure 1:**
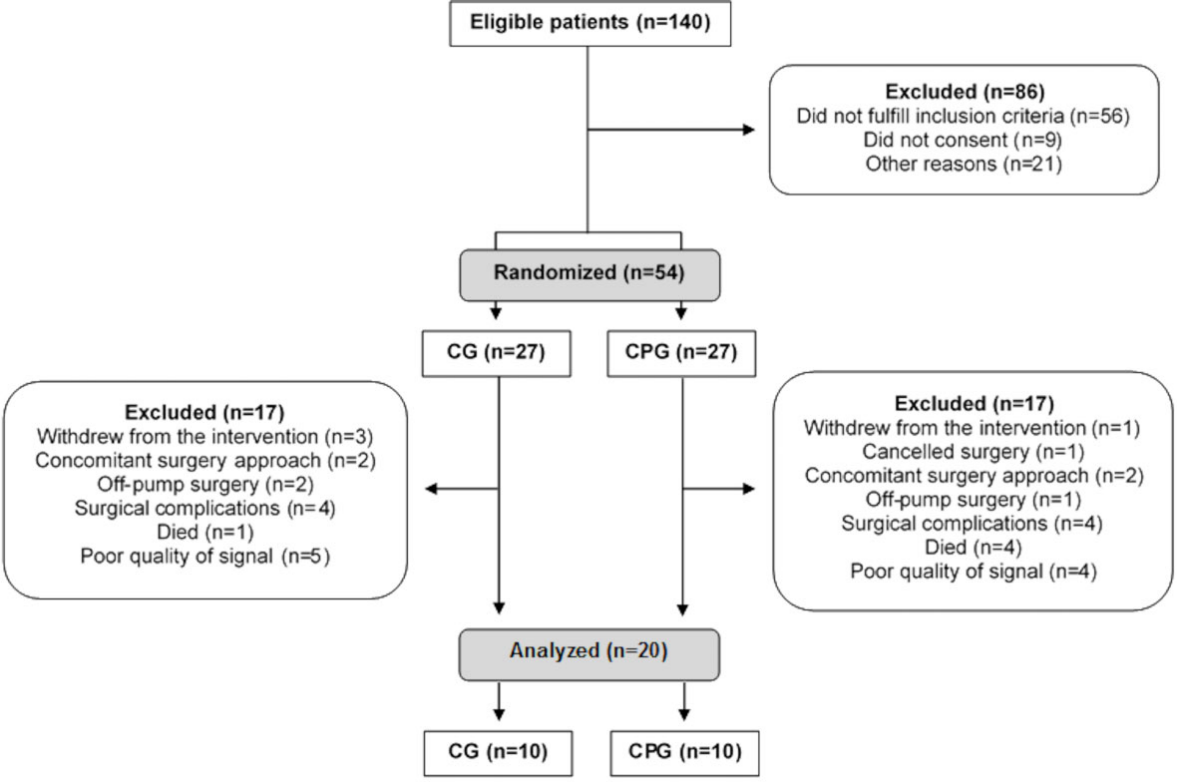
Flowchart showing subjects' participation in the study. n: number of patients; CG: control group; CPG: continuous positive airway pressure group.

Initially, 140 patients were considered eligible, but 86 were excluded due to several factors; thus, 54 patients were randomized into CG (n=27) and CPG (n=27). However, after randomization, 17 patients from each group were excluded, and the final sample was composed by 10 patients in the CG and 10 patients in the CPG (Figure 1). The groups had a similar duration of the protocol (around 5 days).

**Figure 2:**
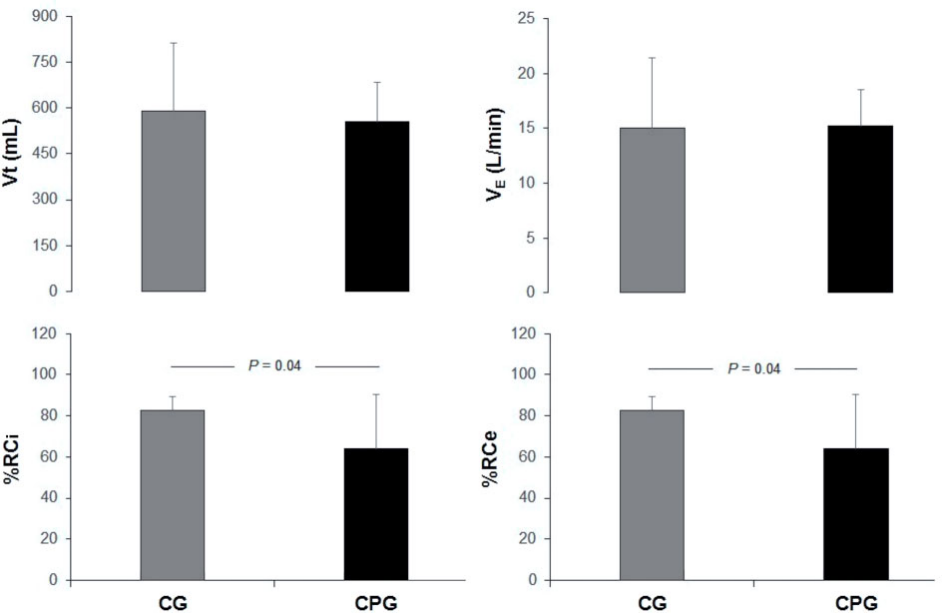
Comparison of the respiratory inductive plethysmography variables of the groups at discharge time. CG: control group; CPG: continuous positive airway pressure group; Vt: tidal volume; V_E_: minute ventilation; %RCi: percent rib cage inspiratory contribution to tidal volume; %RCe: percent rib cage expiratory contribution to tidal volume. Data are reported as means±SD (n=10/group). The unpaired Student's *t*-test was used.

Table 2 shows the baseline, perioperative, and postoperative clinical data of groups; no differences were observed between groups in relation to age, number of males, anthropometry, risk factors, medications, and operative clinical data. Table 3 shows the RIP and HRV variables in the preoperative situation and at discharge time and it can be observed that no differences between groups were present in the preoperative situation. Concerning the discharge situation, HRV indexes of both groups were similar. For RIP variables, the values of Vt and V_E_ were similar between groups, but %RCi and %RCe were significantly lower in the CPG (Figure 2).

**Table 2:** Baseline, perioperative, and postoperative clinical data of groups.

Variables	CG (n=10)	CPG (n=10)	P
Age (years)	55.8±6.1	58.3±8.4	0.46
Males (n)	8	4	0.17
Anthropometry			
Height (m)	1.66±0.09	1.60±0.09	0.21
Weight (kg)	70.9±10.8	70.1±12.0	0.87
BMI (kg/m^2^)	25.8±3.0	27.4±4.7	0.38
Risk factors (n)			
Smoking history	9	6	0.30
Hypertension	8	8	1.42
Diabetes mellitus	5	5	1.34
Medications (n)			
Beta-blockers	10	8	0.47
ACE inhibitor	7	6	1.00
Calcium antagonists	1	-	1.00
Operative clinical data			
CPBT (min)	47.5±16.5	60.5±18.5	0.12
ACCT (min)	28.5±12.0	33.2±10.8	0.37
Coronary artery grafts (n)	2.1±0.6	2.4±0.7	0.31

Data are reported as means±SD or absolute value. CG: control group; CPG: continuous positive airway pressure group; n: number; BMI: body mass index; ACE: angiotensin-converting enzyme; CPBT: cardiopulmonary bypass time; ACCT: aortic cross-clamping time. The unpaired Student's *t*-test and the Fisher's exact test were used for statistical analyses.

**Table 3:** Respiratory inductive plethysmography and heart rate variability data during resting condition in preoperative (Pre) and discharge situation (Post) for both groups.

	CG (n=10)			CPG (n=10)		
	Pre (means±SD)	Post (mean±SD)	P value intragroup	Pre (mean±SD)	Post (mean±SD)	P value intragroup
RIP variables						
Vt (mL)	550.5±151.4	591.5±222.6	0.48	574.6±152.8	557.5±127.5	0.78
V_E_ (L/min)	10.4±3.5	15.0±6.4	0.02	12.8±4.0	15.3±3.3	0.09
%RCi	88.0±9.3	82.6±6.9	0.17	81.1±11.1	63.9±26.3*	0.05
%RCe	87.9±9.2	82.9±6.4	0.16	80.6±11.5	64.0±26.2*	0.06
HRV variables						
Linear HRV						
Mean RR, ms	898.9±127.6	613.0±100.5	<0.01	836.8±144.2	628.9±76.8	<0.01
SDNN, ms	14.6±6.1	6.2±4.1	<0.01	12.7±6.8	6.6±3.6	0.02
Mean HR, bpm	68.0±9.8	100.4±16.9	<0.01	73.7±12.5	96.8±12.5	<0.01
rMSSD, ms	15.8±9.5	6.7±4.8	0.03	13.6±8.2	8.0±4.4	0.08
Non-linear HRV						
SD1, ms	11.2±6.7	4.8±3.4	0.03	9.6±5.8	5.7±3.1	0.08

Data are reported as means±SD. CG: control group; CPG: continuous positive airway pressure group; n: number; RIP: respiratory inductive plethysmography; Vt: tidal volume; V_E_: minute ventilation; %RCi: percent rib cage inspiratory contribution to tidal volume; %RCe: percent rib cage expiratory contribution to tidal volume; HRV: heart rate variability; RR: R-R intervals; SDNN: standard deviation of all N-N normal intervals; HR: heart rate; rMSSD: square root of the mean squared differences of successive RR; SD1: standard deviation of Poincaré plot perpendicular to the line-of-identity. *P<0.05 CPG *vs* CG in Post situation (unpaired Student's *t*-test). P value intragroup: paired Student's *t*-test. No differences were observed between the groups in the preoperative situation.

We also performed an intragroup analysis (preoperative × post rehabilitation) for both groups (Table 3) and observed that %RCi and %RCe variables in the CG were similar between the situations. For the CPG, these variables were lower at discharge time compared to the preoperative situation and this difference, although not statistically significant, probably due to the small sample size (P values of %RCi and %RCi were 0.05 and 0.06, respectively), demonstrated that CPAP patients improved their pattern compared to the preoperative period, which did not happen in the control group. Concerning the HRV variables, both groups presented lower values at discharge time, compared to the preoperative, as expected. In the delta values (post - pre situation) comparison analyses between groups, no significant differences were observed for the RIP and HRV variables.

## Discussion

Our study aimed to evaluate the influence of long-term use of CPAP, associated to the exercises, on BP and HRV. Intervention consisted of a physiotherapy protocol of standardized and progressive exercises associated or not with CPAP as an adjunct of all physical exercises. Our findings indicated that the group who received a CPAP application had a better BP at discharge time compared to those without noninvasive ventilation. Concerning cardiac autonomic modulation, both groups presented decreased HRV indices after surgery and at discharge time, as expected by the impact of surgery on cardiac autonomic modulation (7).

Many studies have demonstrated that the impairment in pulmonary function after CABG is inevitable (19-21) and may be associated to negative outcomes and complications, such as pulmonary edema, pleural effusions, atelectasis, and pneumonia (22). Moreover, the perioperative and postoperative requirements of the surgical procedure (cardiopulmonary bypass, thoracotomy, and pleural drainage) can affect the progression of rehabilitation during hospitalization, contributing to prolonged immobilization.

For this reason, the importance of early inpatient rehabilitation is emphasized in the literature and different sorts of protocols have been studied and applied (10,11,13,23-25). Noninvasive ventilation has also been recognized as an important adjunct to physiotherapy, which is applied at rest (10) and during exercises (5,15) after cardiac surgery, with a variety of outcomes reported. However, studies that examine the long-term effects of CPAP after CABG on BP are still needed.

To the best of our knowledge, the present study is the first to include the CPAP during all the physical exercises of a rehabilitation program and to evaluate its long-term effect on BP. In this context, we demonstrated that patients who received CPAP were discharged with lower values of %RCi and %RCe, which represent a lower upper ribcage inspiratory and expiratory contribution to Vt.

It is known that rib cage and abdomen contributions to the Vt vary on each breath, that rib cage and abdomen compartments are in phase in normal respiration, and the measurement of their expansion can be used to assess respiratory effort (26). Moreover, the movements of the chest and abdominal wall during breathing can vary according to the conditions studied, age, sex, and posture adopted (10,27). However, although some conditions contribute to an increase in the pulmonary rib cage or abdominal excursion (such as verticalization and horizontalization, respectively), a high predominance of one compartment motion may indicate a decrease in the other compartment performance and may be associated with asynchrony breathing (28). Thus, a higher ribcage contribution to Vt can reflect a lower contribution of the abdomen, which can indicate a diminished diaphragmatic activity.

Some authors have studied the ribcage and abdominal excursion during different conditions, confirming an increase in abdominal motion during diaphragmatic breathing. Lage et al. (29) evaluated the chest wall volumes during some breathing conditions in patients with heart failure, including inspiratory-loaded breathing associated with diaphragmatic breathing and observed that when diaphragmatic breathing was associated with inspiratory loaded breathing, a higher volume in the abdominal compartment was achieved, without significant changes in other BP variables.

Vieira et al. (30) studied the influence of quiet breathing and four breathing exercises (diaphragmatic breathing, inspiratory sighs, sustained maximal inspiration, and intercostal exercise) on BP and thoracoabdominal motion in healthy subjects. They observed an increase in abdominal contribution during diaphragmatic breathing, with a consequent decrease of the rib cage contribution to Vt.

Few studies have analyzed the BP during noninvasive ventilation. Our group demonstrated the acute effect of CPAP on BP after CABG (10,15), with positive results of this adjunct in thoracoabdominal synchrony. Soilemezi et al. (31) studied the acute effects of CPAP of 10 cmH_2_O on diaphragmatic kinetics and BP of healthy individuals. Using RIP, they studied the rib cage and abdominal compartment (the latter reflecting diaphragmatic motion). They observed an increase in Vt with CPAP due to an increase in the rib cage contribution to this Vt and no significant change in abdominal motion and abdominal contribution to Vt. According to the authors, the CPAP in these healthy volunteers leads to lung overdistention and recruitment of respiratory muscles, which could increase the energy cost.

However, in the context of post CABG surgery, in which mechanical disadvantages are present, with ventilatory mechanics and BP alterations, our results are especially important, considering that our patients, who received CPAP throughout the whole rehabilitation program, were discharged with a better BP, which could indicate more synchronized breathing. Moreover, these patients were also discharged with a better BP compared to the preoperative period. A possible mechanism for the results of our study may be related to the effects of CPAP, which include respiratory musculature unloading and decrease in physiologic work of breathing, with a greater diaphragmatic performance during the respiratory cycle that, although more pronounced acutely, could persist in the long term.

In a previous study (15), we demonstrated that CPAP acutely improved exercise tolerance and exertion perception on the first day of walking, whereby patients presented better BP, with lower values of %RCi and %RCe, probably due to a decrease in the work of breathing. Although we think that the most significant effect of CPAP may occur in an acute application, mainly considering the first days after surgery, our results demonstrated that patients submitted to a protocol including CPAP throughout the whole rehabilitation program may benefit with a more synchronized breathing, which could also influence outpatient exercise tolerance and performance. However, we did not evaluate the patients after discharge to assess if the benefits of CPAP on BP persisted for a longer time and were associated with other outcomes. Nevertheless, the improvement of BP was a favorable result, especially considering that the CPAP patients improved their pattern compared to the preoperative period, which did not happen in the control group.

Regarding the HRV outcomes, we did not observe differences between the groups concerning the cardiac autonomic modulation at discharge time. Both groups presented significant alterations in cardiac autonomic function after the surgery and were discharged with similar values of variables representative of sympathetic and parasympathetic modulation. We hypothesized that the CPAP can only acutely modify cardiac autonomic modulation, with no alterations in the long term. Moreover, both groups performed a similar amount of physical exercises, which has the potential to positively influence HRV already demonstrated (13).

Our study had some limitations. We were unable to blind the physical therapist to the type of treatment of both groups; however, the analyst was blinded as to the treatment group. Moreover, we did not analyze the subject in the immediate postoperative period to verify the effect of the CR on the outcome variables. We also did not evaluate the patients after discharge to assess if the effect of CPAP on the BP persisted for a longer time. Thus, we are unable to speculate if the BP acquired during the inpatient phase with the CPAP treatment could hasten outpatient rehabilitation. Lastly, the small sample size was an important limitation of our study and we think that future studies with a greater number of participants are necessary.

In conclusion, the results of our study demonstrated that patients of post-CABG surgery who received CPAP application during all the exercises of an inpatient CR program were discharged with a better BP, which could indicate more synchronized breathing. Our findings may have important clinical implications, mainly considering the respiratory mechanical disadvantage after the surgery. CPAP did not influence cardiac autonomic modulation in the long term. Further research regarding this issue is warranted.
